# Identification of small RNAs in *Mycobacterium tuberculosis*

**DOI:** 10.1111/j.1365-2958.2009.06777.x

**Published:** 2009-07-10

**Authors:** Kristine B Arnvig, Douglas B Young

**Affiliations:** Division of Mycobacterial Research, MRC National Institute for Medical ResearchLondon NW7 1AA, UK.

## Abstract

In spite of being one of our most prominent bacterial pathogens, the presence of small regulatory RNAs (sRNAs) has not previously been investigated in *Mycobacterium tuberculosis.* Post-transcriptional regulation of gene expression by sRNA molecules has been demonstrated in a wide range of pathogenic bacteria and has been shown to play a significant role in the control of virulence. By screening cDNA libraries prepared from low-molecular weight RNA from *M. tuberculosis* we have identified nine putative sRNA molecules, including *cis*-encoded antisense transcripts from within open reading frames and *trans*-encoded transcripts from intergenic regions. sRNAs displayed differential expression between exponential and stationary phase, and during a variety of stress conditions. Two of the *cis*-encoded sRNAs were associated with genes encoding enzymes involved in lipid metabolism, *desA1* and *pks12*. These sRNAs showed complementarity to multiple *M. tuberculosis* genes, suggesting the potential to act as both *cis*-encoded and *trans*-encoded sRNAs. Overexpression of selected *trans*-encoded sRNAs had profound impact on growth of *M. tuberculosis* and *M. smegmatis*. This is the first experimental evidence of sRNAs in *M. tuberculosis* and it will be important to consider the potential influence of sRNA regulation when studying the transcriptome and the proteome of *M. tuberculosis* during infection.

## Introduction

*Mycobacterium tuberculosis*, the causative agent of tuberculosis, is one of the most successful human pathogens. It is estimated that a third of the world's population has been infected, with new infections occurring at a rate of approximately one per second. Less than 10% of infected individuals go on to develop active disease, resulting in around 9 million new cases and 1.6 million deaths annually. The remaining 90% will not develop any symptoms but the bacteria may persist in the form of an asymptomatic latent infection with the potential to reactivate at any time.

While the physiological state of *M. tuberculosis* during active or latent infection remains poorly understood, considerable efforts have been invested in exploring transcriptional changes associated with bacterial adaptation to changing environments in *in vitro* and cell culture models. The genome of *M. tuberculosis* encodes 13 sigma factors, 11 two-component sensory transduction systems, and over 100 annotated transcriptional regulators (Cole *et al*., 1998); mutagenesis and transcriptional profiling studies have implicated many of these in the response to stress stimuli and during pathogenesis in experimental models.

There is growing evidence in other bacteria that the mechanisms for transcriptional control are complemented by a post-transcriptional regulatory network dependent on small regulatory RNA (sRNA) molecules, which can act by base pairing or by interaction with proteins (Waters and Storz, 2009). Most of the known base pairing sRNAs can be divided into two categories: *cis*-encoded sRNAs that are transcribed in the antisense orientation to their target protein-encoding mRNA, and *trans*-encoded sRNAs that are transcribed from intergenic regions, and which often have multiple targets (Vogel and Wagner, 2007; Waters and Storz, 2009). The latter category sometimes require the help of the RNA chaperone Hfq depending on the species (Bohn *et al*., 2007; Vogel and Wagner, 2007; Vogel, 2009). sRNA binding can enhance or repress translation of mRNA targets by base-pairing with the 5′ end of the mRNA at different locations relative to the ribosome binding site and start codon, or can alter mRNA stability by generating duplex molecules which act as substrates for RNase III or RNase E (Gottesman, 2004; 2005; Vogel *et al*., 2004). Numerous sRNAs have been described in *Escherichia coli* and it is estimated that the genome may encode several hundred sRNAs (Vogel and Sharma, 2005). Regulation by sRNAs has been implicated in stress responses, in the bacterial cell cycle, and also in the virulence of a range of enteric pathogens, *Listeria monocytogenes* and *Staphylococcus aureus* (Romby *et al*., 2006; Livny and Waldor, 2007; Padalon-Brauch *et al*., 2008). The aim of the present study was to investigate the occurrence of sRNAs in *M. tuberculosis*.

Two general approaches have been taken to identify sRNA molecules in bacteria (Vogel and Sharma, 2005). The first involves bioinformatic prediction by sequence alignment of intergenic regions with known sRNAs, together with identification of appropriately positioned signals for transcriptional initiation and termination (e.g. (Argaman *et al*., 2001; Wassarman *et al*., 2001; Livny *et al*., 2005; 2006). The relatively poor definition of transcriptional signals in mycobacteria led us to take the alternative approach of direct analysis of low-molecular-weight RNA molecules isolated from *M. tuberculosis* cultures (RNomics, e.g. Vogel *et al*., 2003; Kawano *et al*., 2005). We describe a set of nine putative sRNAs identified by this approach and characterized by Northern blotting and transcript mapping by 5′ and 3′ RACE analysis.

## Results

### Cloning of small RNAs from *M. tuberculosis*

Previous reports have identified the two small structural RNAs, tmRNA and RNaseP RNA from *M. tuberculosis* (Svard *et al*., 1996; Mignard and Flandrois, 2007); however, no regulatory RNAs have been identified to date. Therefore our initial experiment was aimed at determining the actual presence and abundance of small transcripts, regarded as putative sRNAs. Total RNA from exponential and stationary growth phases was depleted of rRNA, and labelled with ^32^P-pCp and RNA ligase. The RNA was separated on a denaturing acrylamide gel and visualized by phosphorimaging. Multiple abundant and well-defined small transcripts were observed, with a difference in the pattern of expression between the two growth phases (Fig. S1). This prompted us to proceed with further experiments.

As we had observed differential expression of *M. tuberculosis* small transcripts, two independent cDNA libraries were generated from exponential and stationary phase cultures. Total RNA was size fractionated, and transcripts between 20 and 75 nucleotides were eluted from the gel. The eluted RNA was tailed with CTP and an RNA linker was attached to the 5′ end. The RNA was subsequently converted into cDNA, PCR amplified, cloned and sequenced. A total of 192 clones were sequenced and these fell into six categories according to the TubercuList webserver (http://tuberculist.epfl.ch): 1: mRNA fragments within open reading frames (ORFs) (11 clones); 2: rRNA spacer fragments (5 clones); 3: tRNA fragments (1 clone); 4: unknown RNA encoded in intergenic regions (*trans* candidates, 23 clones representing six unique regions and 19 repeat clones, see below), named according to the original library clones; 5: unknown RNA encoded antisense to annotated ORFs (*cis* candidates, 4 clones), named according to their antisense partner; 6: fragments that could not be assigned to a single region due to small size (< 17 base pairs) or chimeric sequence (149 clones). The intergenic regions containing B11 and F6 were represented 16 and three times respectively, and in both growth phases, indicating that transcripts from these regions were relatively abundant.

**Fig. 1:**
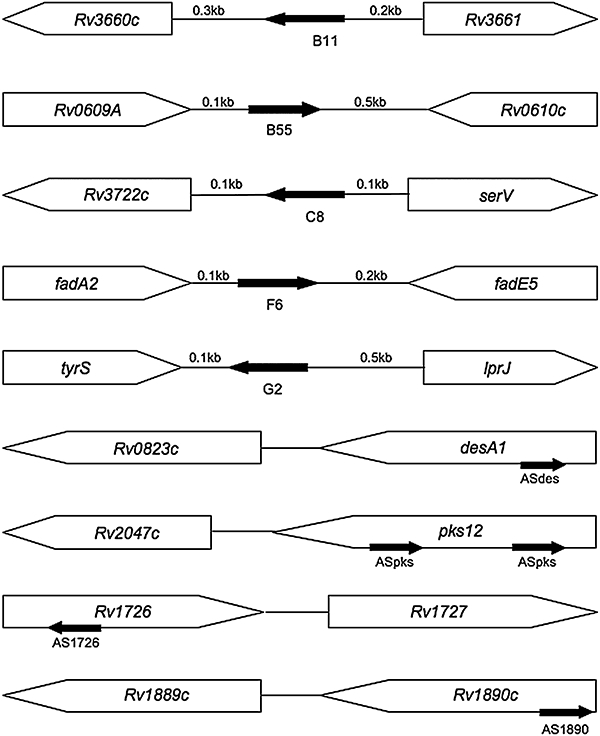
Genomic positions of sRNA candidates. Schematic showing genome locations of the four *cis*- and five *trans*-encoded sRNA candidates as determined by sequence analysis of the cDNA library clones. sRNAs are shown as black arrows with names of sRNA candidates. The *pks12* gene contains two identical copies of the region encoding ASpks. Approximate distances between cloned sequences and ORF beginnings (*cis* candidates) or flanking ORFs (*trans* candidates) are indicated.

The genomic positions and context of the unknown RNAs were identified in order to determine the likelihood of each to be a genuine sRNA or part of the adjacent gene (Fig. 1). If an intergenic cDNA clone was encoded on the opposite strand of an adjacent gene, or more than 100 base pairs from an adjacent gene on the same strand, it was considered a valid sRNA candidate. Based on these criteria most of the intergenic clones appeared to be independent transcripts and not 5′ or 3′ UTRs. One clone was located 5 base pairs upstream of *Rv 2050* and therefore considered to be the 5′ UTR of this gene and thus not investigated further. The sizes and genome locations of the remaining sRNA candidates are shown in Table 1.

**Table 1:** *M. tuberculosis trans*-encoded sRNAs.

sRNA	Clone(s) (size)	Northern (∼size)	5′ end^a^	3′ end^b^	Transcript (calculated size^c^)	Left gene	Right gene
B11	69	(70), 85	4099478	4099386^U^	**93**	*Rv3660c*; essential Cons. hypothetical	*Rv3661* Cons. hypothetical
B55	37	55	704187	704247	**61**	*Rv0609A* CT^d^Cons. hypothetical	*Rv0610c* hypothetical
C8	29	85–140	4168281	4168154^U^4168212 4168224	58, 70, 128	*Rv3722c* CT essential	tRNA-Ser (*serV*)
F6	30	55, 100	**293604**	293641 293661 293705^U^	38, **58**, **102**	*Rv0243*/*fadA2* CT Acetyl-CoA acyl transferase	*Rv0244c/fadE5* Acyl-CoA dehydrogenase
G2	57	65	**19151901915028**	1914962 1914977	**67**, 214, 229	*Rv1689*/*tyrS* tyrosine tRNA synthase; essential	*Rv1690 /lprJ* lipoprotein

sRNA	clone(s) (size)	Northern (∼size)	5′ end^a^	3′ end^b^	Transcript (calculated size^c^)	Complementary gene(s)	

ASdes	30	75, 110	**918264918350918365**	918432 918412 918458	48, 63, **68**, 83, 94, **109**, 149, 169, 195	*desA1/Rv0824c*; essential fatty acid desaturase (*desA2/Rv1094*; essential fatty acid desaturases)^e^	
ASpks	54	75, (200)^f^	2299745 **22997852299796**	2299886 2299873	**78**, 89, 91, 102, 129, 142, 162	*pks12/Rv2048c*; polyketide synthase (PDIM synthesis) (*pks7, pks8, pks15;* polyketide synthases)^e^	
AS1726	27	80	**19525031952400**1952375 1952367 1952351	1952291	61, **77**, 85, 110, 213	*Rv1726*; possible oxireductase	
AS1890	36	110	2139419 2139466 2139548 2139594	2139656	63, **109**, 191, 238	*Rv1890c*; unknown hypothetical	

a Listed are all 5′ ends determined by library clones and 5′ RACE; 5′ ends in bold indicate transcription start sites according to RACE.

b 3′ ends are determined by RACE and/or by scrutiny in which case the position corresponds to the first U in the terminator ^U^.

c Transcript sizes show all the possible sizes from the 5′-and 3′ ends listed; figures shown in bold correspond to sizes seen on Northern blots.

d CT indicates co-transcribed with sRNA. Genes listed as essential are according to predictions from transposon mutagenesis.

e Additional genes that could in theory interact with sRNA.

f Transcript only seen during certain stress conditions.

All co-ordinates shown are according to the TubercuList webserver; co-ordinates for ASpks are for one of two identical sRNA encoding regions.

### Northern blots verify the presence of sRNAs in *M. tuberculosis*

**Fig. 2:**
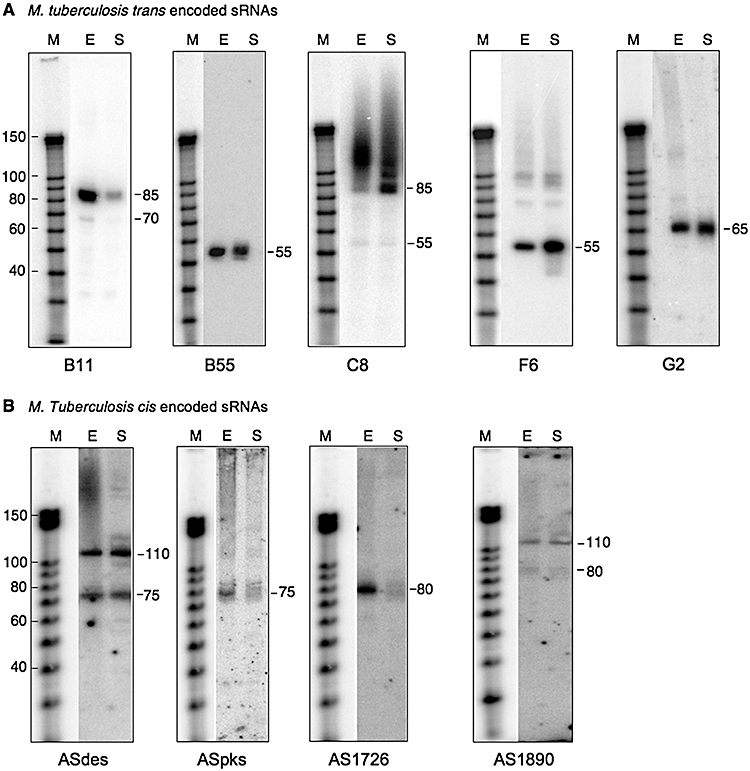
Northern blots verify the presence of *M. tuberculosis* sRNAs. RNA from exponential (E) and stationary phase (S) cultures was analysed by Northern blotting using riboprobes complementary to cDNA clones identified as sRNA candidates; A shows *trans*-encoded sRNAs; B shows *cis*-encoded sRNAs. Transcript sizes are approximate and compared with Ambion's Decade marker (M).

Our cDNA libraries identified nine sRNA candidates; five *trans*-encoded (B11, B55, C8, F6 and G2) and four *cis*-encoded [antisense (AS) to *desA1*, *pks12*, *Rv 1726* and *Rv 1890c*]. To further clarify if the identified candidates were genuine sRNAs, we performed Northern blotting with riboprobes complementary to the original cDNA clones. Before hybridization the membranes were stained with methylene blue to ensure uniform loading between exponential and stationary phase RNA (not shown). The results of the Northern blotting, shown in Fig. 2, demonstrate signals corresponding to small transcripts from each of the candidates. Judging from the signals on the Northern blots the *trans*-encoded sRNAs were expressed at significantly higher levels than the *cis*-encoded ones. All transcripts (except a faint ∼70-nucleotide signal from B11 exponential phase) were larger than the cloned fragments, suggesting that the latter were probably degradation products. The majority of sRNAs were seen as more than one transcript, and most of the transcripts appeared to be differentially expressed and/or processed in the two growth phases. The ASdes and ASpks blots showed a significant smear representing larger species (> 150 nucleotides), specifically in exponential growth, suggesting the presence of larger transcripts/precursors in addition to the small transcripts. The C8 sRNA appeared as a large smear with the majority of the signal between 110 and 140 nucleotides in exponential phase, less so in stationary phase where a distinct band around 85 nucleotides was seen, suggesting extensive trimming of the primary transcript to the 85-nucleotide RNA. In addition there was a faint band around 55 nucleotides. A summary of the approximate sizes of transcripts found by Northern blotting is shown in Table 1.

### Mapping of transcripts

**Fig. 3:**
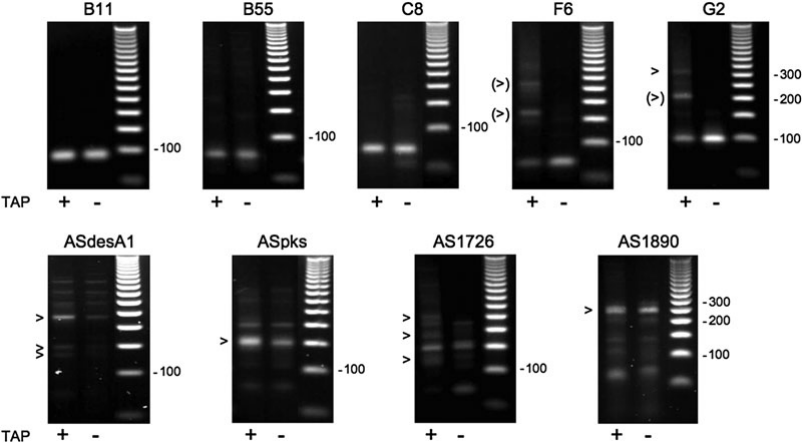
Mapping of 5′ ends with RACE. RLM-RACE analysis was used to map the 5′ ends of sRNA transcripts in TAP (+) and Mock (–) treated samples. Transcription start sites (indicated by arrows) were identified on the basis of a stronger signal in TAP-treated samples. Sequence analysis of TAP-dependent bands indicated by bracketed arrows in F6 and G2 identified them as concatamers with 5′ sequences identical to original cDNA clones.

sRNA transcripts were further characterized by RNA ligase-mediated rapid amplification of 5′ and 3′ complementary DNA ends (RLM-RACE). The 5′ ends were mapped by comparing the RACE products obtained with and without prior treatment with tobacco acid pyrophosphatase (TAP), which facilitates the differentiation of transcription start sites from processed 5′ ends (Argaman *et al*., 2001). By hydrolysing the terminal 5′-triphosphate group found only in primary transcripts, TAP enables the ligation of a 5′ linker to these transcripts thereby enhancing the signal from transcription start sites (see *Experimental procedures* for details). The results, shown in Fig. 3, suggest several putative transcription start sites, indicated by arrows. Bands representing significant 5′ ends were excised, cloned and sequenced. As the Northern blots in most cases indicated differential expression at different stages of growth, we also performed 5′ RACE on RNA from stationary phase (not shown). A summary of the identified 5′ ends is given in Table 1. Together the results indicate that some of the RNAs contain a single distinct and seemingly processed 5′ end (B11, B55, C8). F6 and G2 as well as all the *cis*-encoded sRNAs appeared to have several different 5′ ends, primary as well as processed, of varying intensity. However, some of the larger products from the F6 and G2 reactions turned out to be concatemers of transcripts with the same 5′ end as the library clones. As the concatemerization was TAP dependent we consider these 5′ ends to be transcription start sites. This means that G2 was the only *trans*-encoded sRNA with more than one 5′ end, while all of the *cis*-encoded RNAs had multiple 5′ ends.

**Fig. 4:**
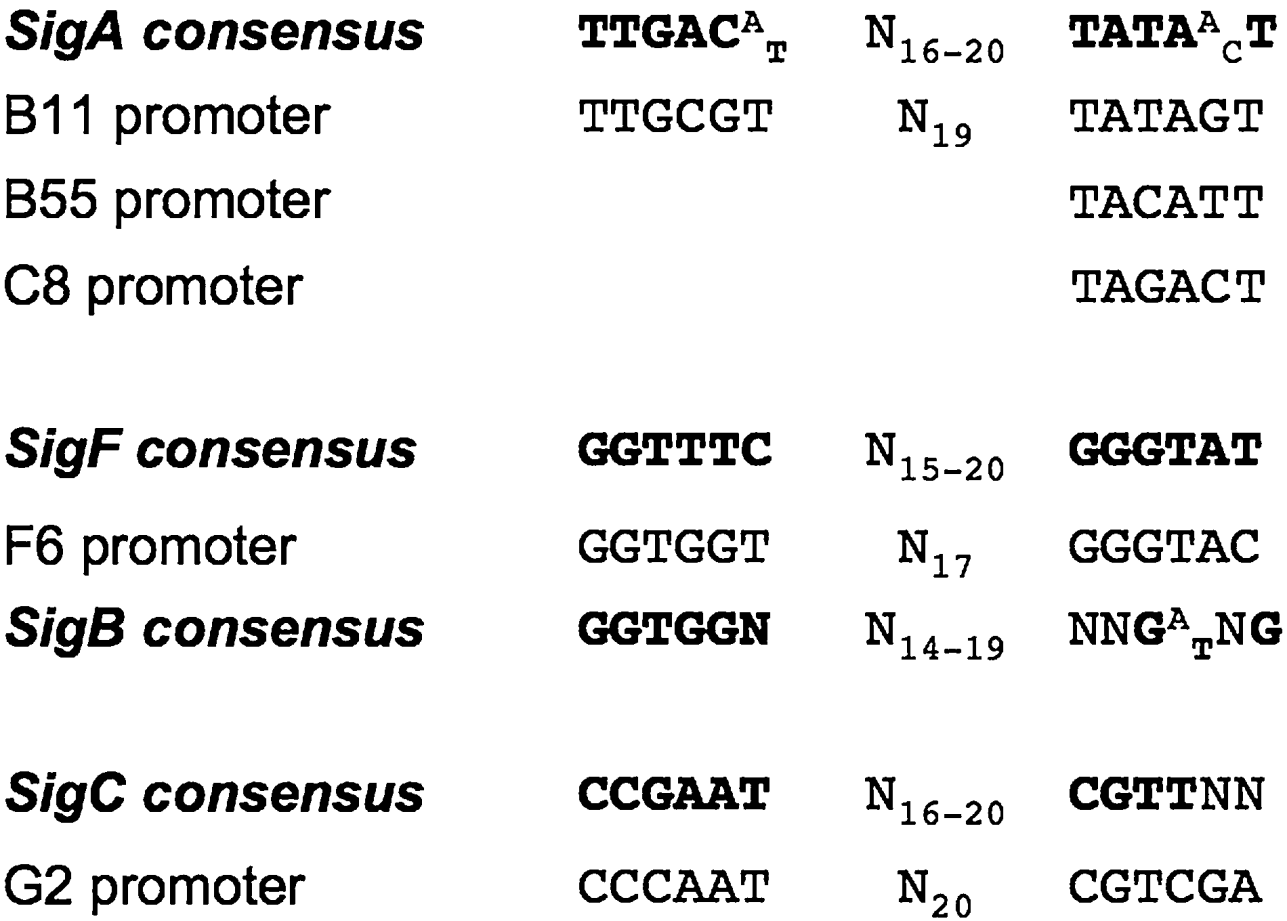
Alignment of putative sRNA promoters with *M. tuberculosis* promoter consensus sequences. Putative promoter motifs, found immediately upstream of the identified 5′ ends of the *trans*-encoded sRNAs, have been aligned to promoter motifs of the most likely consensus sequence.

In the case of B11 we identified a putative SigA promoter immediately upstream of the 5′ end suggesting that this may be a transcription start site (Fig. 4).

We also identified a putative SigF promoter upstream of the F6 5′ end as well as slightly weaker similarities to the SigB consensus, suggesting that this promoter could be recognized by both sigma factors. In the case of G2 we identified a possible SigC promoter upstream of one of the 5′ ends. The close homology of these sequences to the shown consensus sequences strongly support the notion of the identified 5′ ends being transcription start sites. Due to the heterogeneity of *M. tuberculosis* promoters the assignment of promoter sequences for the remaining transcription start sites were somewhat more uncertain. However, we did identify putative −10 boxes upstream of C8 and B55, but no −35 boxes. The −10 region upstream of the C8 5′ end was identical to the −10 box of the PCL1 promoter of the *M. tuberculosis rrn* operon, recognized by SigA. The situation was similar for B55, which displayed some homology to the −10 region of a SigA promoter but no identifiable −35 region (Fig. 4).

As the RACE experiments did not indicate any transcription start sites specific for stationary phase, we conclude that the observed differences in sizes between the two growth phases were due to differential processing, rather than different promoter usage.

We were able to identify likely terminators, consisting of a stem-loop followed by a poly-U stretch, for B11, C8 and F6. These were located 92, 127 and 101 nucleotides downstream of their respective 5′ ends (including the first U of the poly U tail). In the case of B11 this would result in a 93-nucleotide transcript in reasonably good agreement with the Northern blot. The situation for C8 was not as straightforward and hence we performed 3′ RACE to verify the C8 3′ end (see below). In the case of F6, a 102-nucleotide transcript is in good accordance with the faint signal of approximately 100 nucleotides seen on the Northern blot. This transcript could then be processed to the shorter and more abundant ∼55-nucleotide transcript.

In order to map the remaining 3′ ends, we performed 3′ RACE. A poly-A tail was added to the RNA and 3′ RACE was carried out as described in *Experimental procedures*. In some cases an additional 3′ RACE was performed on poly-C-tailed RNA for comparison. The 3′ ends thus identified were compared with the 5′ ends and the transcript sizes on the Northern blots in order to identify accurate transcript boundaries. Results of 3′ RACE analysis are summarized in Table 1. In the case of C8 this approach suggested two additional 3′ ends, which resulted in transcript sizes of 70 and 58 nucleotides respectively. One scenario is that transcription of C8 ends at the suggested terminator resulting in a 128-nucleotide transcript, with progressive 3′ trimming generating the shorter transcripts. However, the smear on the Northern blot suggests additional larger C8 species, indicating more distal promoters and/or terminators. Some of the sRNAs contained start codons (ATG and/or GTG) within the transcript boundaries, but most of the stop codons, for these reading frames, were located downstream of the mapped 3′ ends. However, the AS1726 transcript did contain a small ORF of 99 nucleotides, and it is possible that this transcript encodes a functional protein, although we could not identify a likely ribosome binding site.

### Transcriptional coupling and sequence conservation

Apart from G2 all of the *trans* encoded sRNA candidates were encoded on the same strand as one of the adjacent protein-encoding genes (Fig. 1). This prompted us to test by RT-PCR if these sRNAs were co-transcribed with the particular upstream or downstream gene. Using primers that spanned the sRNA and the adjacent gene, we found that B55 was co-transcribed with the upstream *Rv0609A* (Fig. S2). Together with the fact that the region between *Rv0609A* and B55 is 100% conserved (in *M. tuberculosis* complex only), this suggests the possibility that B55 is part of the 3′ UTR of the *Rv0609A* mRNA rather than being an sRNA. Although we had identified two 3′ ends for C8 by RACE, as well as a possible terminator between C8 and the downstream (essential) *Rv3722c*, we found that C8 was co-transcribed with *Rv3772c* (Fig. S2). This would explain the presence of the larger C8 species (seen as a smear) on the Northern blot and furthermore suggests that termination at the proposed terminator may not be 100% efficient. Sequence comparisons with other bacterial genomes showed that the first 90 nucleotides of C8 are highly conserved in mycobacteria as well as more distantly related bacteria, i.e. *Rhodococcus*, *Corynebacteria* and *Nocardi*a. Searching the Rfam database (Griffiths-Jones *et al*., 2005; Gardner *et al*., 2009) with the C8 sequence revealed that this RNA was in fact 4.5S RNA, which is known to be trimmed extensively (Li *et al*., 1998), but which has not previously been annotated in *M. tuberculosis.* F6 was found to be co-transcribed with the upstream *fadA2*. F6 itself was found to be highly conserved in pathogenic mycobacteria, while the 3′ half was less well-conserved in non-pathogenic mycobacteria (Fig. S3). No RT-PCR product was obtained using primers spanning B11 and *Rv3660c*, indicating that these two genes are not co-transcribed and therefore B11 is a bona fide sRNA. The sequence of B11 was highly conserved in a wide range of pathogenic mycobacteria as well as in the non-pathogenic *Mycobacterium smegmatis* (Fig. S3). Finally G2 was conserved only within the *M. tuberculosis* complex.

We also tested if the genes for which we had identified *cis*-encoded sRNAs, were co-transcribed with their downstream genes, because there was less than 100 base pairs between these ORFs. The results indicate that all of the presumed target genes were co-transcribed with a downstream partner. This arrangement suggests that the *cis*-encoded sRNAs have the potential for post-transcriptional modulation of the ratio between target gene and its downstream partner (Fig. S2).

### Prediction of secondary structure of sRNAs

Once we had identified the 5′ and 3′ termini of the transcripts we assigned the most probable boundaries for the transcripts seen on the Northern blots. Each of the resulting sequences was subsequently analysed using *mfold* for prediction of secondary structures (Mathews *et al*., 1999; Zuker, 2003). Strikingly all of the sequences, except B55 and C8 (4.5S) had a C:G ratio > 1 (Fig. S4). In particular, the loops/single-stranded regions of the predicted structures often contained unpaired, C-rich stretches analogous to *S. aureus* RNAIII (Novick *et al*., 1993; Benito *et al*., 2000). Of the *trans*-encoded sRNA candidates, B55 was the only predicted structure devoid of such a C-rich loop. The sequences corresponding to *M. smegmatis* B11 and F6 was also subjected to this procedure, and the resulting structures were almost identical to the *M. tuberculosis* structures (not shown).

### Expression of *M. tuberculosis* sRNAs during stress

**Fig. 5:**
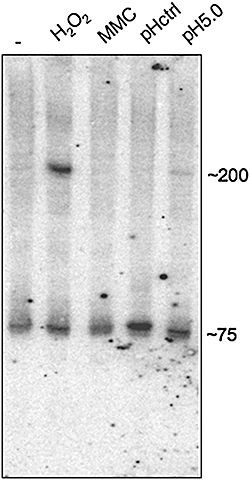
Northern blot of *M. tuberculosis* ASpks sRNA after stress. The blot illustrates of the appearance of a novel ASpks transcript upon induction with 10 mM H_2_O_2_. Lanes: exponential (–); 2: H_2_O_2_ treated; 3: Mitomycin C treated (MMC); 4: pH control; 5: acid stress (pH 5.0).

Bacterial sRNAs are often stress induced. We wanted to investigate how the expression of *M. tuberculosis* sRNAs responded to stresses similar to those encountered during infection. Therefore, we subjected cultures of *M. tuberculosis* to oxidative stress (induced by H_2_O_2_), DNA damage (induced by Mitomycin C) and acid stress. Total RNA was isolated and sRNA expression detected by Northern blotting and subsequently normalized to 5S RNA levels to compensate for uneven loading. If an sRNA had multiple bands, we quantified all the bands. Each signal was compared with a control sample, i.e. unstressed, exponential growth for oxidative stress and DNA damage, and a pH control for the acid stress (see *Experimental procedures* for details). A representative Northern blot for each sRNA upon stress is shown in Fig. S5, and diagrams showing each sRNA's average response to stress are shown in Fig. S6. From this experiment it was evident that stress-induced expression varied significantly between sRNAs, with some remaining unchanged and others displaying more than twofold increase upon stress. Oxidative stress was the most potent inducer of differential expression with fold-induction ratios of 1.8, 2.4, 2.0 and 2.5 for B11, B55, F6 and ASpks respectively (Table S2). The increase in ASpks expression was associated with the striking appearance of a novel ∼200-nucleotide transcript that we had not observed previously (Fig. 5). In addition, F6 showed a twofold induction in response to acid stress. Reproducible quantification of C8 (i.e. 4.5S RNA) proved difficult due to the transcript showing up as a smear rather than distinct bands.

### Overexpression of *M. tuberculosis* sRNAs

**Fig. 6:**
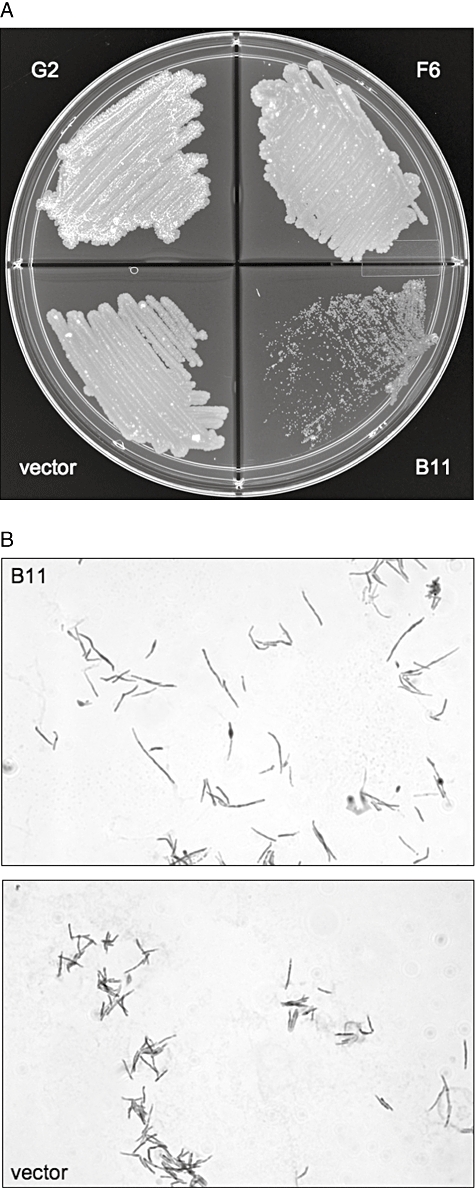
Overexpression of *M. tuberculosis* sRNAs in *M. smegmatis*. A illustrates the growth on solid 7H11 of *M. smegmatis* expressing B11, F6, G2 compared with empty vector. B shows representative slides of stained *M. smegmatis* expressing B11 compared with cells with empty vector. Cells were grown overnight, resuspended in PBS + 5% Tween 80 and stained with carbolfuchsin.

In order to gain some insight into the function of *trans*-encoded sRNAs, B11, F6 and G2 were cloned in plasmid vectors under the control of the strong *rrnB* promoter of *M. smegmatis* (Arnvig *et al*., 2005) and transformed into *M. tuberculosis* and *M. smegmatis mc*^*2*^*155*. The constructs expressing B11 and G2 both proved lethal in *M. tuberculosis,* while the expression of F6 resulted in extremely slow growth with pin prick colonies visible after 3–4 weeks. Constructs with B11 and G2 driven by a weaker promoters also led to cell death. The expression of B11 in *M. smegmatis* had a pronounced phenotype with very slow growth on plates and in liquid medium (Fig. 6A and data not shown). Staining revealed that the cells were elongated sometimes with lumps or protrusions (Fig. 6B), suggesting that B11, which is highly conserved between *M. tuberculosis* and *M. smegmatis* (Fig. S5), is involved in some aspect of cell wall synthesis and/or cell division. *M. smegmatis* expressing G2 also showed defective growth, especially in liquid medium (not shown), in spite of the absence of any obvious homologue of this sRNA in the rapid-growing strain. The expression of F6 did not result in any apparent growth defects in *M. smegmatis*, although the 5′ end and central, loop-containing part of this sRNA is conserved (Fig. S3).

## Discussion

We have shown here for the first time experimental evidence of sRNAs in *M. tuberculosis*. By screening cDNA libraries prepared from low-molecular-weight *M. tuberculosis* RNA, we cloned nine sRNAs which were all readily visualized by Northern blotting and were further characterized by mapping of 5′ and 3′ ends. All of the cloned transcripts appeared to be degradation products of the native sRNAs, judging from size differences detected by Northern blot. All of the *M. tuberculosis* sRNAs display stable predicted secondary structures and in most cases a C:G ratio > 1. They include transcripts from intergenic regions (*trans*-encoded sRNAs) as well as short antisense transcripts encoded within ORFs (*cis*-encoded sRNAs). One of the *trans*-encoded sRNAs was identified as the *M. tuberculosis* homologue of the 4.5S RNA molecule that forms part of the signal recognition particle involved in protein secretion (Driessen and Nouwen, 2008).

Although the percentage of putative sRNAs clones that we obtained (13%) was high in comparison with that reported using a similar approach with RNA from *E. coli*, i.e. 3% (Kawano *et al*., 2005), we presume that we have detected only a subset of the total repertoire of *M. tuberculosis* sRNAs. Preliminary results of sequence-based RNomic screens reveal multiple potential sRNA signals for *M. tuberculosis* in addition to our initial set of transcripts (K.B. Arnvig and N.R. Thomson, unpubl. obs.).

A recent bioinformatic screen in *Streptomyces*, a related actinomycete, predicted 32 sRNAs, of which 20 were confirmed experimentally (Panek *et al*., 2008), while a similar screen using sRNAPredict2 predicted 56 sRNAs in *M. tuberculosis* (Livny *et al*., 2006). None of our experimentally isolated sRNAs were predicted by this method. This could be due to the heterologous nature of the *M. tuberculosis* promoters as well as the lack of canonical terminator structure, which were part of the search parameters in their study. We do note, however, that Livny *et al*. did not predict B11, which displays sequence homology to SigA (i.e. sigma-70 like) promoters as well as having a canonical terminator structure.

Neither *Mycobacteria* nor *Streptomyces* have identifiable homologues of the Hfq RNA chaperone that has been widely implicated in the mechanism of *trans*-encoded sRNAs in Gram-negative bacteria (Sun *et al*., 2002). Alternative pathways may be important for sRNA regulation in GC-rich Gram-positive bacteria. *M. tuberculosis* sRNAs contain C-rich stretches similar to those found in *Streptomyces* (Panek *et al*., 2008). One function of these regions could be initiating the interaction between sRNA and target mRNA, as seen in the case of *S. aureus* RNA III, which, incidentally, does not require Hfq for function (Geisinger *et al*., 2006; Bohn *et al*., 2007). Alternatively, it may be that C-rich stretches are recognized by an alternative RNA chaperone that functions in place of Hfq, analogous to the recognition of similar sequences by eukaryotic RNA-binding proteins (Makeyev and Liebhaber, 2002).

Northern blot analysis demonstrated variations in sRNA expression between exponential and stationary growth phases, and in response to environmental stress. Three of the *trans*-encoded sRNAs were significantly induced by oxidative stress, for example, while the *cis*-encoded ASpks was expressed at a higher level and in the form of a longer transcript. These findings are consistent with previous reports of a role for sRNAs in bacterial responses to stress e.g. (Gottesman *et al*., 2006), and suggest that sRNAs could be involved in adaptation of *M. tuberculosis* to conditions encountered during infection, as it is the case for other pathogens (e.g. Romby *et al*., 2006; Toledo-Arana *et al*., 2007).

**Fig. 7:**
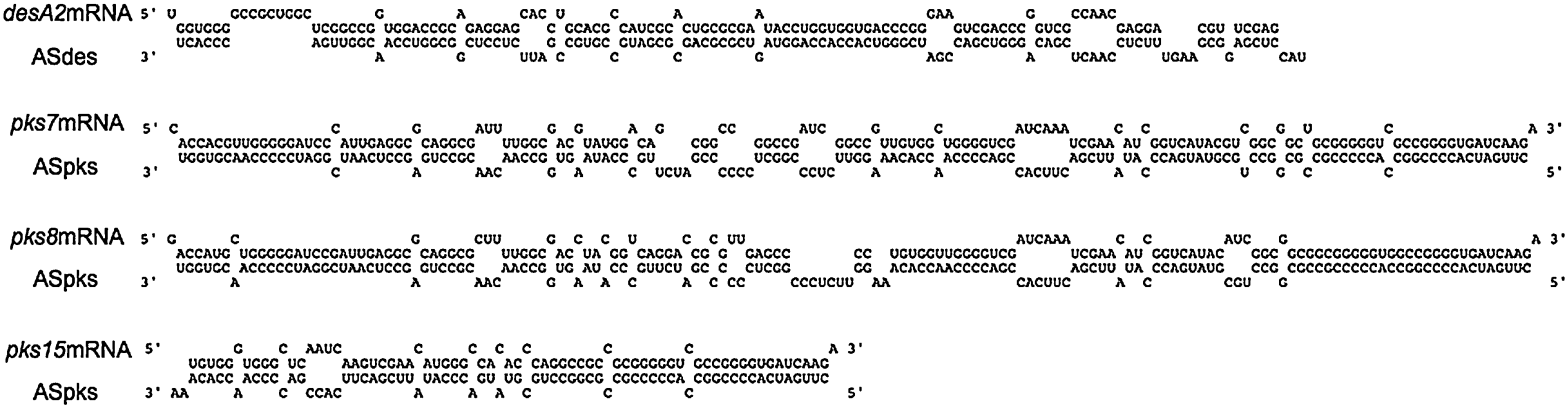
*cis*-encoded sRNA with *trans*-acting potential. Diagram illustrating the extent of complementarity between ASdes and ASpks and their respective alternative targets, *desA2* and *pks7, 8* and *15*.

While there has been a rapid increase in identification of bacterial sRNAs over the last few years, the identification of mRNA and protein targets has proceeded more slowly (Vogel and Wagner, 2007). We can begin to speculate about the potential role of the *cis*-encoded sRNAs in *M. tuberculosis* by examining the function of their antisense partners. Interestingly, the two target ORFs with known function –*desA1* and *pks12*– are both involved in lipid metabolism. The fatty acid desaturase DesA1 (*Rv0824c*) is an essential gene for growth of *M. tuberculosis* (Sassetti *et al*., 2003) and is strongly upregulated following uptake by macrophages and during experimental infection in mice (Schnappinger *et al*., 2003). ASdes shows significant complementarity to a second desaturase (*desA2*/*Rv1094*) (Fig. 7), and could therefore regulate expression of both genes, acting as both a *cis*-encoded and a *trans*-encoded sRNA. ASpks is expressed as an antisense transcript within the *pks12* gene, the largest ORF in *M. tuberculosis*. Polyketide synthase (Pks) 12 is implicated in synthesis of mannosyl-β-1-phosphomycoketide molecules, which are recognized as antigens by CD1-restricted T cells (Matsunaga *et al*., 2004). ASpks aligns with mRNA encoding duplicate identical ketosynthase domains between amino acid residues 404–458 and 2427−2481 (Gokhale *et al*., 2007). Similar ketosynthase domains are present in of *pks7*, *pks8* and *pks15*, giving ASpks the potential to act as both *cis*-encoded and *trans*-encoded sRNA depending on the boundaries of the sRNA (Fig. 7).

It is more difficult to speculate on potential targets and functions for the *trans*-encoded sRNAs, and the possibility remains that some of these could be structural rather than regulatory RNAs, as was the case of 4.5S RNA, meaning they would not have mRNA targets as such. However, genome and operon associations with genes of known function may give a clue. Thus, F6 is located within a region encoding enzymes involved in lipid metabolism as well as co-transcribed with *fadA2*, for which the transcript is downregulated in macrophages (Schnappinger *et al*., 2003). Phenotypic changes resulting from experimental manipulation of sRNA expression levels provide an alternative route to target identification. Overexpression of three *trans*-encoded sRNAs had profound effects on mycobacterial growth. Strikingly, no colonies were obtained for *M. tuberculosis* following transformation with B11, while *M. smegmatis* transformants had major defects affecting cell division and morphology. Overexpression of G2 also prevented growth of *M. tuberculosis* and reduced growth of *M. smegmatis*, while F6 reduced growth specifically in *M. tuberculosis*. These experiments demonstrate that sRNAs have a functional role in *M. tuberculosis*. We are currently following up on these observations using regulated expression systems in combination with transcriptome profiling to identify targets and mechanisms for *trans*-encoded sRNA regulation.

In summary, our results provide the first demonstration that *M. tuberculosis* expresses sRNA molecules and that these play an important functional role in bacterial physiology. We anticipate that further functional studies in combination with sequence-based RNomics will provide novel insights into the fundamental biology of tuberculosis with the potential to inform development of improved strategies for disease control.

## Experimental procedures

### Strains and growth conditions

*Escherichia coli* DH5α was grown on Luria–Bertani agar with 50 μg ml^−1^ kanamycin. *M. tuberculosis* laboratory strain H37Rv was grown in Middlebrook 7H9 medium with 10% ADC in roller bottle culture. Exponential phase cultures were harvested at OD between 0.5 and 0.8; stationary phase cultures were harvested 1 week after OD had reached 1.0. *M. smegmatis* was grown in Middlebrook 7H9 with 10% ADC and 20 μg ml^−1^ kanamycin.

### Stress stimuli

To induce oxidative stress, 30% H_2_O_2_ was added to an exponentially growing culture (OD∼0.6) to a final concentration of 10 mM for 1 h. To induce DNA damage, Mitomycin C was added to an exponentially growing culture (OD∼0.3) at a final concentration of 0.02 μg ml^−1^ for 24 h. Acid stress was induced by spinning down an exponentially growing culture at room temperature and resuspending in acidified medium (pH 5.0) at 37°C to OD 0.3–0.4 and subsequently grown for 24 h. In order to take into account the hypoxia and cold shock induced during centrifugation, a control culture was treated identically but resuspended in standard 7H9 medium (pH 6.5) and likewise grown for 24 h and used for normalizing the acid stressed samples.

### Plasmid construction

The overexpression vector was made by replacing the XbaI-HindIII fragment containing the Hsp60 promoter in pMV261 (Stover *et al*., 1991) with the XbaI-HindIII fragment spanning −200 to −8 of the *rrnB* promoter from *M. smegmatis* (Arnvig *et al*., 2005). The vector was made such that the sRNA could be inserted as a HindIII fragment downstream of the −10 region, and such that transcription would start at the mapped +1 with none or one added nucleotide at the 5′ end. A synthetic transcriptional terminator was made by annealing oligos Term.F and Term.R (Table S1) and inserted at the HindIII site downstream of the sRNA-3′ end in case the sRNA terminator was not included or not sufficient. The sRNA inserts were made by PCR (B11) or oligo annealing (F6 and G2) using the oligos listed in Table S1. Plasmids were transformed into mycobacteria by electroporation.

### Oligonucleotides

The sequences of all RNA and DNA oligonucleotides used for cDNA libraries, probe construction, RACE and RT-PCR are listed in Table S1.

### RNA isolation and handling

Cultures were cooled rapidly by the addition of ice directly to the culture before centrifugation. RNA was isolated by means of the FastRNA Pro blue kit from QBiogene/MP Bio according to manufacturer's instructions. Before ^32^P-pCp-labelling the RNA was depleted of 16S and 23S rRNA by means of the microbExpress kit from Ambion according to instructions.

### Labelling of RNA

Depleted RNA was end-labelled with ^32^P-pCp (GE Healthcare) and RNA ligase (Ambion) according to instructions. Riboprobes were made with Ambion's mirVana miRNA Probe construction kit using the cDNA cloned sequence as template (Table S1) and ^32^P-UTP (800 mCi mmol^−1^; GE Healthcare).

### cDNA library construction

Total RNA from the two growth phases was separated and poly-C-tailed using *E. coli* poly(A) polymerase (Ambion) and CTP. The RNA was treated with TAP and a 5′ linker (Table S1) was added according to instructions (Generacer kit, Invitrogen). RNA was reverse transcribed with a mix of oligo(dG) primers (GRdG3′, Table S1) and subsequently PCR amplified with the same oligo(dG) primer mix and a linker specific primer (GR5′, Table S1) using PuReTaq (GE Healthcare). PCR products were cloned into pCRII-TOPO (Invitrogen) and sequenced.

### Northern blotting

Total RNA was separated on 8% or 10% denaturing acrylamide gels and electroblotted onto Ambion's Brightstar membranes. After UV cross-linking the membranes were stained in 0.3 M sodium acetate containing 0.03% methylene blue. The membranes were incubated overnight with riboprobes in UltrahybOligo and exposed to phosphorimaging after washing. Stress-induced changes in RNA expression were determined by densitometer-scanning of Northern blots. Transcript sizes were compared with Ambion's Decade marker (10–150 nucleotides) and RNA marker low from Abnova (20–500 nucleotides).

### RACE

5′ RACE was carried out essentially as described by Argaman *et al*. (2001), except the reverse transcription was carried out using random primers and Superscript III (Invitrogen). PCR amplification of cDNA was performed with PuReTaq using GR5′ (linker specific, Table S1) and genes-specific reverse primers (Table S1). PCR reactions were separated on 3.5% NuSieve agarose (Lonza) and bands of interest were excised, cloned into pCR-II TOPO and sequenced.

3′ RACE was performed by adding a poly(A) or poly(C) tail to total RNA with *E. coli* poly-A-polymerase (Ambion), reverse transcribing with Superscript III (Invitrogen) using the GRdT oligo or GRdG oligo (Table S1) and finally PCR amplifying with PuReTaq (GE Healthcare) using GR3′ and gene-specific forward primers (Table S1).

### RT-PCR

cDNA was made with random primers and Superscript II (Invitrogen). A control reaction was carried out in parallel without enzyme. A standard PCR reaction was subsequently carried out with primers listed in Table S1.
