# Supplementary material for: Identification of small RNAs in Mycobacterium tuberculosis
Source: Mol Microbiol. 2009 Jul 10;73(3):397–408. doi: 10.1111/j.1365-2958.2009.06777.x (PMC2764107; doi:10.1111/j.1365-2958.2009.06777.x)
Supplement: Supplementary file 1 [file mmi0073-0397-SD1.pdf]

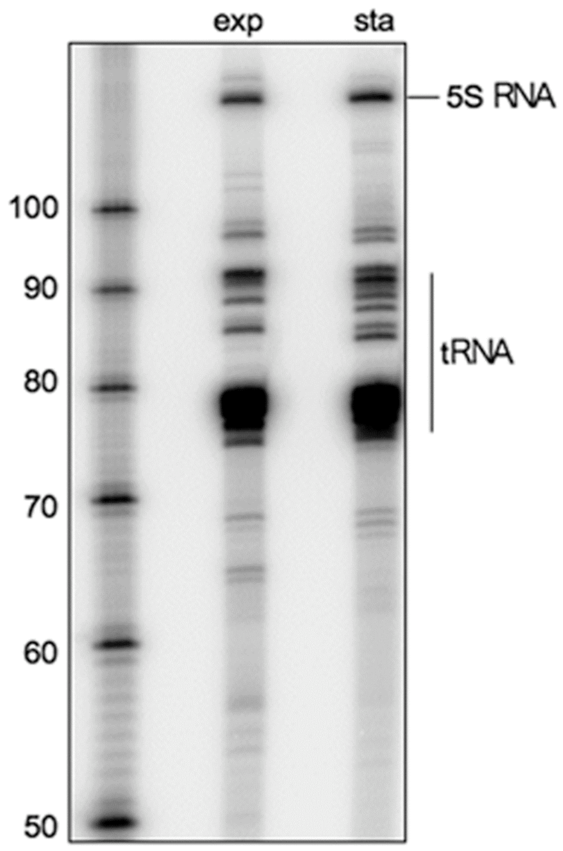

Fig. S1. Small transcripts are differentially expressed in exponential and stationary phase cultures of *M. tuberculosis*

RNA extracted from exponential (exp) and stationary (sta) phase cultures was depleted for rRNA, radio-labelled at the 3’ end, and analysed by acrylamide gel electrophoresis and phosphorimager. Size marker is shown in the left lane.

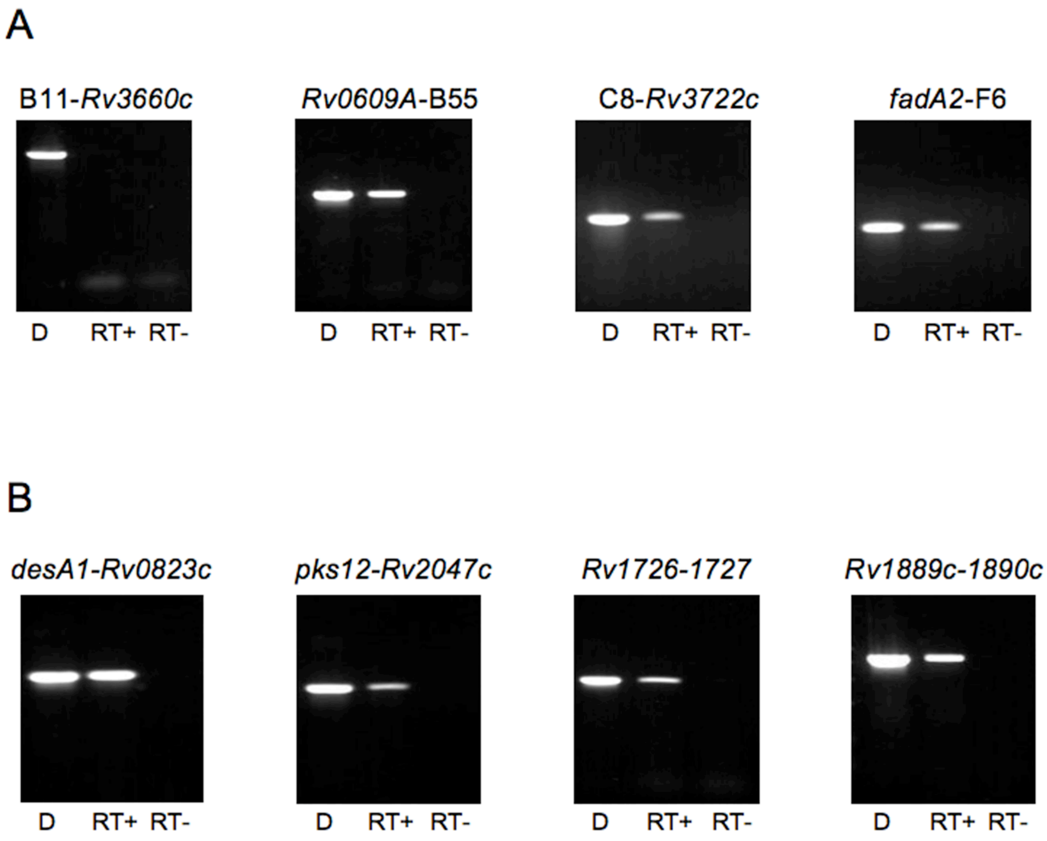

Fig. S2. RT-PCR analysis of transcriptional coupling

A illustrates results of RT-PCR analysis of transcriptional coupling between *trans*-encoded sRNA candidates and adjacent ORFs. B55, F6 and C8 were found to be co-transcribed with ORF-encoding mRNAs; B11 was present on a separate transcript. (G2 is transcribed in the opposite orientation to both flanking ORFs). D, DNA control; RT+, reverse transcription in the presence of reverse transcriptase; RT-, reverse transcription in the absence of reverse transcriptase

B illustrates RT-PCR analysis to explore whether ORFs containing *cis*-encoded sRNAs are co-transcribed with adjacent ORFs. Demonstration of a single transcript in each case suggests the potential for sRNA-dependent modulation of the ratio between target gene and its downstream partner.

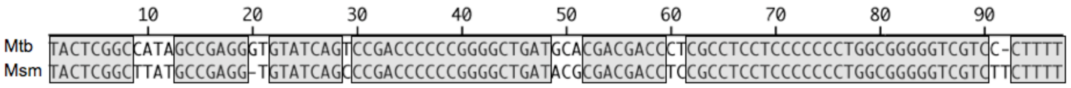

B11

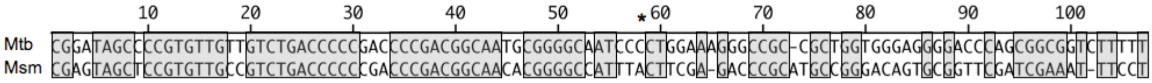

F6

Fig. S3. Sequence conservation of sRNAs

Alignment of the *M. tuberculosis* B11 and F6 sRNAs with their homologues from the non-pathogenic *M. smegmatis*. Identical nucleotides are boxed in grey. The 3' end of *M. tuberculosis* F6, mapped by RACE, is shown by asterisk.

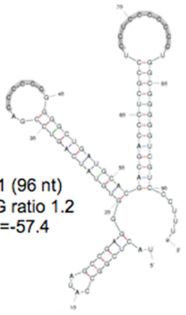

B11 (96 nt)
C:G ratio 1.2
ΔG=-57.4

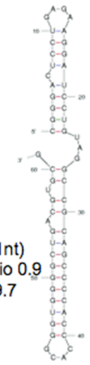

B55 (61nt)
C:G ratio 0.9
ΔG=-39.7

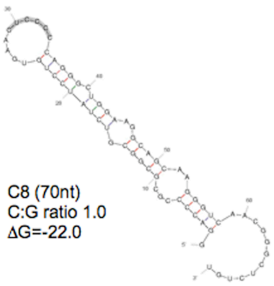

C8 (70nt)
C:G ratio 1.0
ΔG=-22.0

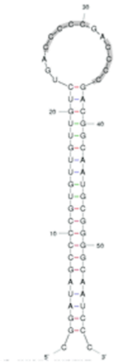

F6 (58nt)
C:G ratio 1.1
ΔG=-32.6

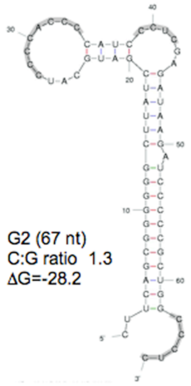

G2 (67 nt)
C:G ratio 1.3
ΔG=-28.2

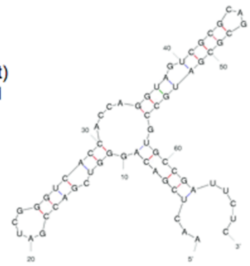

ASdes (68nt)
C:G ratio 1.1
ΔG=-21.3

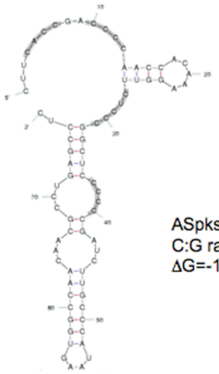

ASpks (78nt)
C:G ratio 1.5
ΔG=-14.9

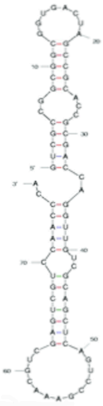

AS1726 (77nt)
C:G ratio 1.1
ΔG=-29.0

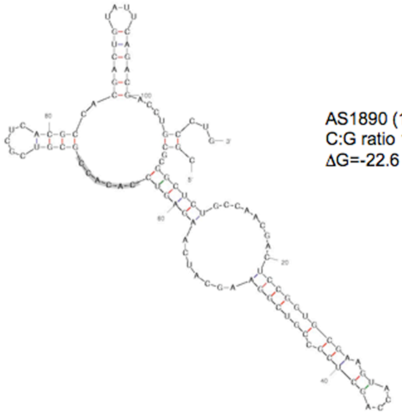

AS1890 (109nt)
C:G ratio 1.1
ΔG=-22.6

Fig. S4. Predicted structures of sRNAs according to *mfold*

Representative examples of predicted sRNA structures, i. e. one for each sRNA. Structure predictions indicate stable, compact molecules with a high degree of secondary structure. C-rich single-stranded regions are highlighted in grey.

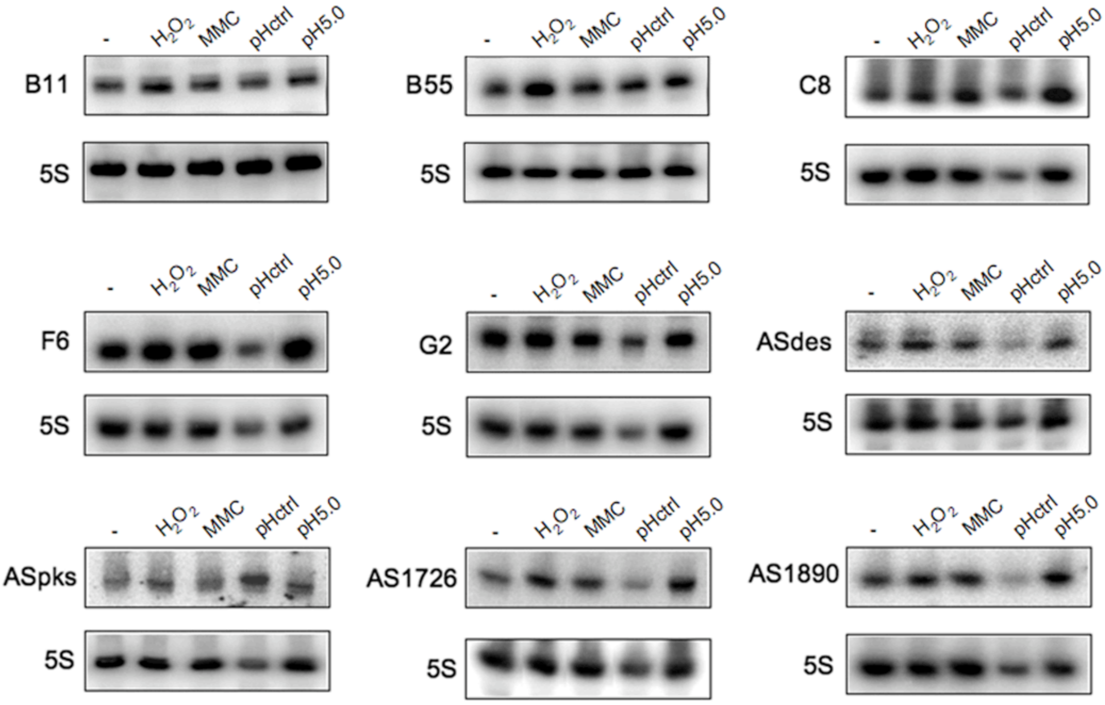

Fig. S5. Stress responses of *M. tuberculosis* sRNAs

The panels show representative Northern blots of RNA isolated from stressed *M. tuberculosis* upon stress. The 5S RNA band used for normalisation is included in each case. Lanes, -: exponentially grown culture; H_2O_2 treated; MMC: Mitomycin C treated; 4: pH control; 5: acid stress, pH 5.0.

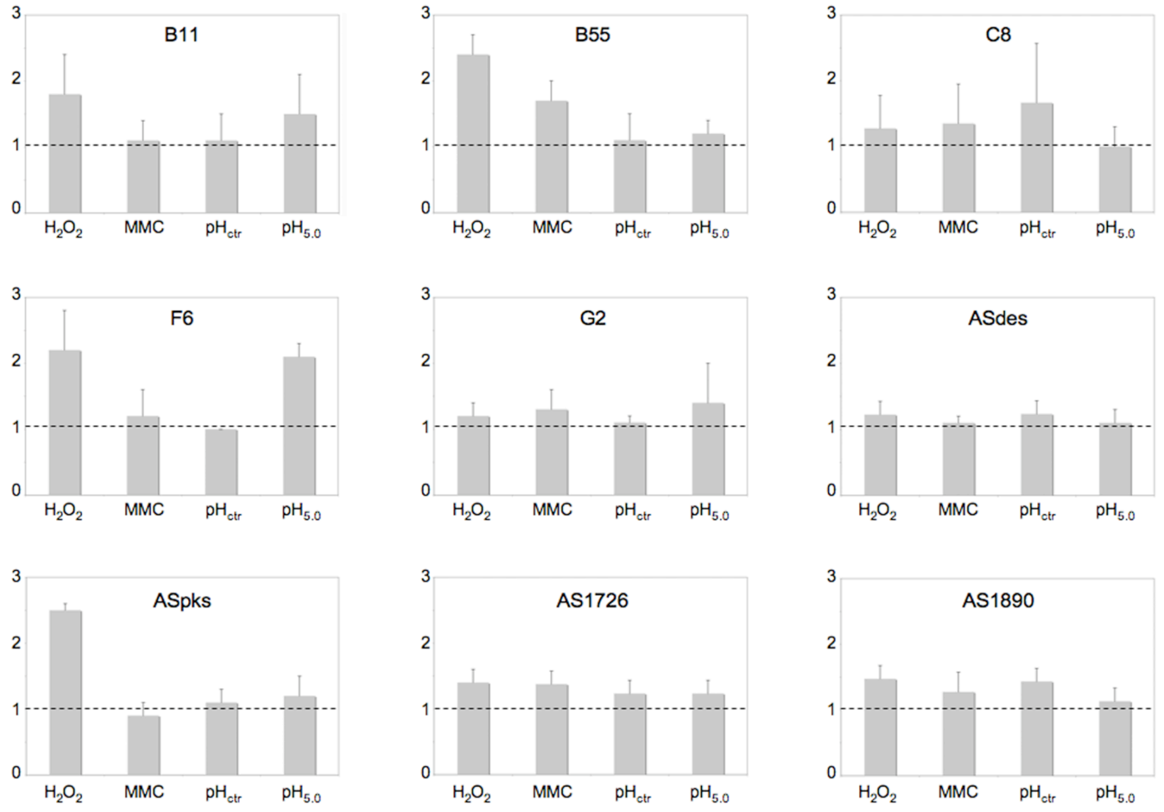

Fig. S6. Stress responses of *M. tuberculosis* sRNAs

Diagrams showing the average changes in expression of each sRNA determined by Northern blotting. Each value represents the mean of three to four independent experiments, normalized to 5S RNA and shown with standard deviation. The value of each sample is normalised to the appropriate control samples (i. e. H_2O_2 and Mitomycin C (MMC) are relative to exponential growth and pH 5.0 is relative to pH control).

Table S1: oligos used in this study

**Oligos for library construction**	
RNA 5' linker	CGACUGGAGCACGAGGACACUGACAUGGACUGAAGGAGUAGAAA
GRdG3'A/C/G/T	GCTGTCAACGATACGCTACGTAACGGCATGACAGT(G)10A/C/G/T
GR5'	CGACTGGAGCACGAGGACACTGA
GR3'	GCTGTCAACGATACGCTACGTAACG
**Template oligos for Riboprobes***	
B11-nrt	GGCCATAGCCGAGGGTGTATCAGTCCGACCCCCCCTGTCTC
B55-nrt	CGGGACTCCTGAGAAGGATCCTGTAGGCCGCAGCCTGTCTC
C8-nrt	GGACCCCGCGCGGCGTCTATCCTGTGAACCTGTCTC
F6-nrt	CGGATAGCCCCGTGTTGTTGTCTGACCTGTCTC
G2-nrt	ATCGATGCATCCCCACCCCATCCCTCGAGATAAGCCTGTCTC
ASpks-nrt	CAACGCCTGAGCCTCAATCGGATCCCCCAACGTGGCCTGTCTC
ASdes-nrt	TCTCCTCGGCGGTCCACCGGTTGACCCACTCCTGTCTC
AS1726-nrt	AGTCCGAAACGTCGAGTCGTCCAACCCCCTGTCTC
AS1890-nrt	TCACGCCACGACTGTATTCAGACGACCTGCCTGCCTGTCTC
5S-nrt	GTCCCATTCCGAACCCGGAAGCTAAGCCTGCCAGCGCCTGTCTC
**Oligos for RACE**	
GR5'	CGACTGGAGCACGAGGACACTGA
GRdT	GCTGTCAACGATACGCTACGTAACGGCATGACAGTGT(T)24
GR3'	GCTGTCAACGATACGCTACGTAACG
B11.f	GCTGATGCACGACGACCCTCGC
B11.r	CCGGGGGGTCGGACTGATACACC
B55.f	CGGGACTCCTGAGAAGGATCCTGTAGGCCGC
B55.r	CTGCGGCCTACAGGATCCTTCTCAGG
C8.f	CCCCGCGCGGCGTCTATCCTGTG
C8.r	TCACAGGATAGACGCCGCGCGGGG
F6.f	CGGATAGCCCCGTGTTGTTGTCTGACCCCC
F6.r	TCAGACAACAACACGGGGCTATCC
G2.f	CCAGGCTTCAGCGGGGGGCTTATCG
G2.r	CTTATCTCGAGGGATGGGGTGGGGATGC
ASpks.f	GGCCAACAACGCCTGAGCCTC
ASpks.r	GGGACCACGGTGGGGATCCGATTGAG
ASdes.f	GCGCGATGCCGTGCCGATTCTC
ASdes.r	GTCAACCGTTGGACCGCCGAGGAG
AS1726.f	AGTCCGAAACGTCGAGTCGTCCAACCC
AS1726.r	
AS1890.f	TCACGCCACGACTGTATTCAGACGACCTGCCTG
AS1890.r	GGCAGGTCGTCTGAATACAGTCGTGGCGTG
**oligos for RT-PCR**	
RT-B11.F	(same as B11.f)
RT-3660.R	CGCCAGGGCAACCGCAAACAAC
RT-0609.F	GCGGTGCGAGGCTCACGGGTACGACTATTT
RT-B55.R	GGGGCTGCGGCCTACAGGATCCTTCTCAG
RT-C8.F	GCGCGGCGTCTATCCTGT
RT-3722.R	GCCCTCCGGGTCGCGGTAGTCGTCT
RT-fadA2.F	GCGGCGACCGGTGGGCGGATTTT
RT-F6.R	TCGGGGGTCAGACAACAACACGGGGCTATC
RT-desA1.F	CGAGCTGGCCTGCGACAAGT
RT-0823.R	CTGCAGCGAGCGTGGTGATTC
RT-2048.F	CGCCGACCGCAGAACAAGAGAT
RT-2047.R	TTCGGCCACGCACCCACGCACAAT
RT-1726.F	ACGGGCTGGGCACCGCGTATGTGAACTT
RT-1727.R	GCGTCGGCGAGGAGTAGCTGTCGGCTTCTA

Table S1: oligos used in this study (continued)

**Oligos for overexpression plasmids**	
B11.F	AAAAGCTTGGTACTCGGCCATAGCCGAGGGTG
B11.R	TTAAGCTTAAAAGGGACGACCCCCGCCAG
F6.F	AGCTTGGCGGATAGCCCCGTGTTGTTGTCTGACCCCCGACCCCGACGGCAATGCGGGGCAATCCCA
F6.R	AGCTTGGGATTGCCCCGCATTGCCGTCGGGGTCGGGGGTCAGACAACAACACGGGGCTATCCGCCA
G2.F	AGCTTGGCTTCAGCGGGGGGCTTATCGATGCATCCCCACCCCATCCCTCGAGATAAGATCCCCCGCTGGCCCTCA
G2.R	AGCTTGAGGGCCAGCGGGGGATCTTATCTCGAGGGATGGGGTGGGGATGCATCGATAAGCCCCCCGCTGAAGCCA
Term.F	AGCTTCCCCGCGAAAGCGGGGTTTTTTTTTTT
Term.R	AGCTAAAAAAAAAAACCCCGCTTTCGCGGGGA

* The 3'ends of template oligos are complementary to the T7 promoter oligo supplied in probe construction kit

Table S2. Induction fold of *M. tuberculosis* sRNA upon stress

sRNA	H_2O_2	MMC	pH_{ctr}	$pH_{5.0}$*
B11	1.8±0.6	1.1±0.3	1.1±0.4	1.5±0.6
B55	**2.4±0.3**	1.7±0.3	1.1±0.4	1.2±0.2
C8	1.3±0.5	1.3±0.6	1.7±0.9	1.3±0.3
F6	**2.0±0.6**	1.2±0.4	1.0±0.0	**2.1±0.2**
G2	1.2±0.2	1.3±0.3	1.1±0.1	1.4±0.6
ASdes	1.2±0.2	1.1±0.1	1.2±0.2	1.1±0.2
ASpks	**2.5±0.1**	0.9±0.2	1.1±0.2	1.2±0.3
AS1726	1.4±0.2	1.4±0.2	1.1±0.2	1.2±0.2
AS1890	1.5±0.2	1.3±0.3	1.4±0.2	1.1±0.2

Values represent mean of three to four independent experiments shown with standard deviation
Induction folds above two are shown in bold
* Induction fold of $pH_{5.0}$ is relative to pH_{ctr}
